# Host Cell Restriction Factors of Paramyxoviruses and Pneumoviruses

**DOI:** 10.3390/v12121381

**Published:** 2020-12-02

**Authors:** Rubaiyea Farrukee, Malika Ait-Goughoulte, Philippa M. Saunders, Sarah L. Londrigan, Patrick C. Reading

**Affiliations:** 1 Department of Microbiology and Immunology, The University of Melbourne at the Peter Doherty Institute for Infection and Immunity, Melbourne VIC 3000, Australia; philippa.saunders@unimelb.edu.au (P.M.S.); sarahll@unimelb.edu.au (S.L.L.); 2 Roche Pharma Research and Early Development, Roche Innovation Center Basel, 4070 Basel, Switzerland; malika.ait-goughoulte@roche.com; 3 WHO Collaborating Centre for Reference and Research on Influenza, Victorian Infectious Diseases Reference Laboratory at the Peter Doherty Institute for Infection and Immunity, Melbourne VIC 3000, Australia

**Keywords:** pneumovirus, paramyxovirus, innate, restriction factor, replication, interferon-stimulated gene

## Abstract

The paramyxo- and pneumovirus family includes a wide range of viruses that can cause respiratory and/or systemic infections in humans and animals. The significant disease burden of these viruses is further exacerbated by the limited therapeutics that are currently available. Host cellular proteins that can antagonize or limit virus replication are therefore a promising area of research to identify candidate molecules with the potential for host-targeted therapies. Host proteins known as host cell restriction factors are constitutively expressed and/or induced in response to virus infection and include proteins from interferon-stimulated genes (ISGs). Many ISG proteins have been identified but relatively few have been characterized in detail and most studies have focused on studying their antiviral activities against particular viruses, such as influenza A viruses and human immunodeficiency virus (HIV)-1. This review summarizes current literature regarding host cell restriction factors against paramyxo- and pneumoviruses, on which there is more limited data. Alongside discussion of known restriction factors, this review also considers viral countermeasures in overcoming host restriction, the strengths and limitations in different experimental approaches in studies reported to date, and the challenges in reconciling differences between in vitro and in vivo data. Furthermore, this review provides an outlook regarding the landscape of emerging technologies and tools available to study host cell restriction factors, as well as the suitability of these proteins as targets for broad-spectrum antiviral therapeutics.

## 1. Introduction

*Paramyxoviridae* and *Pneumoviridae* are two closely related virus families, comprised of enveloped, negative-strand RNA viruses with a non-segmented genome and include several viruses of importance in regard to human and animal health. Notable human pathogens of the *Paramyxoviridae* family include measles (MeV), mumps (MuV) and parainfluenza viruses (PIV-1, 2, 3) [1] (Table 1). The *Pneumoviridae* family, a previous subfamily of *Paramyxoviridae*, includes human metapneumovirus (HMPV) and human respiratory syncytial virus (HRSV), both of which are respiratory pathogens [2,3]. Infections by human paramyxo- and pneumoviruses are associated with significant disease burden. They induce acute respiratory infection (ARI) consisting of upper respiratory tract infections including rhinopharyngitis and sinusitis, and more severe lower respiratory tract infections (LRTIs) such as bronchiolitis and pneumonia. Infections of the respiratory tract by PIV-3 (and less commonly PIV-1 and 2), HMPV and HRSV are generally associated with mild respiratory symptoms in adults, but can result in severe disease in children, the elderly, and other susceptible groups. In contrast, MuV is associated with systemic infections that result in inflammatory reactions, including swelling of the parotid glands, encephalitis, meningitis, myocarditis, and orchitis [4]. Similarly, MeV infection can affect multiple organs, leading to high fever, rash, conjunctivitis, and photophobia [5]. Moreover, both MeV and MuV are neurotropic, and a rare consequence of persistent MeV infection is a neurogenerative disease called subacute sclerosing panencephalitis [6]. Paramyxoviruses can also cause a wide variety of diseases in animals and birds, and members for this virus family include Newcastle disease virus (NDV), canine distemper virus (CDV), and Riderperst virus, which, among others, are associated with significant disease [7]. Indeed, some viruses, such as NDV, have the potential to cause enormous economic losses through impact on the poultry industry [8]. As paramyxoviruses can infect a diverse array of animals, including pigs, bats, birds, and cows, their zoonotic potential is of concern. Spillover events have been reported where Hendra (HeV) and Nipah viruses (NiV) from horses and fruit bats, respectively, have resulted in human infections, often associated with severe disease [7], highlighting their potential to infect and emerge as novel human pathogens.

While effective vaccines are available for MeV and MuV [39], limited therapeutics are available for HRSV, HMPV, or PIV-3 (Table 1). In recent times, concerns have arisen over the re-emergence of MeV, especially in immunocompromised patients and children, due to reduced vaccine coverage [5]. As new modalities of therapy are being explored for these viruses, their interaction with the innate immune system provides an avenue for host-targeted therapies. Of particular interest are cellular antiviral proteins, also known as host cell restriction factors, which can have a limiting effect on viral replication. This review aims to summarize the current literature on host cell restriction factors identified for paramyxo- and pneumoviruses and to provide perspectives on future research in this area, including considerations regarding their potential as host-targeted therapies to prevent severe disease associated with these viral infections.

## 2. Infection of Host Cells and Viral Replication

**Figure 1:**
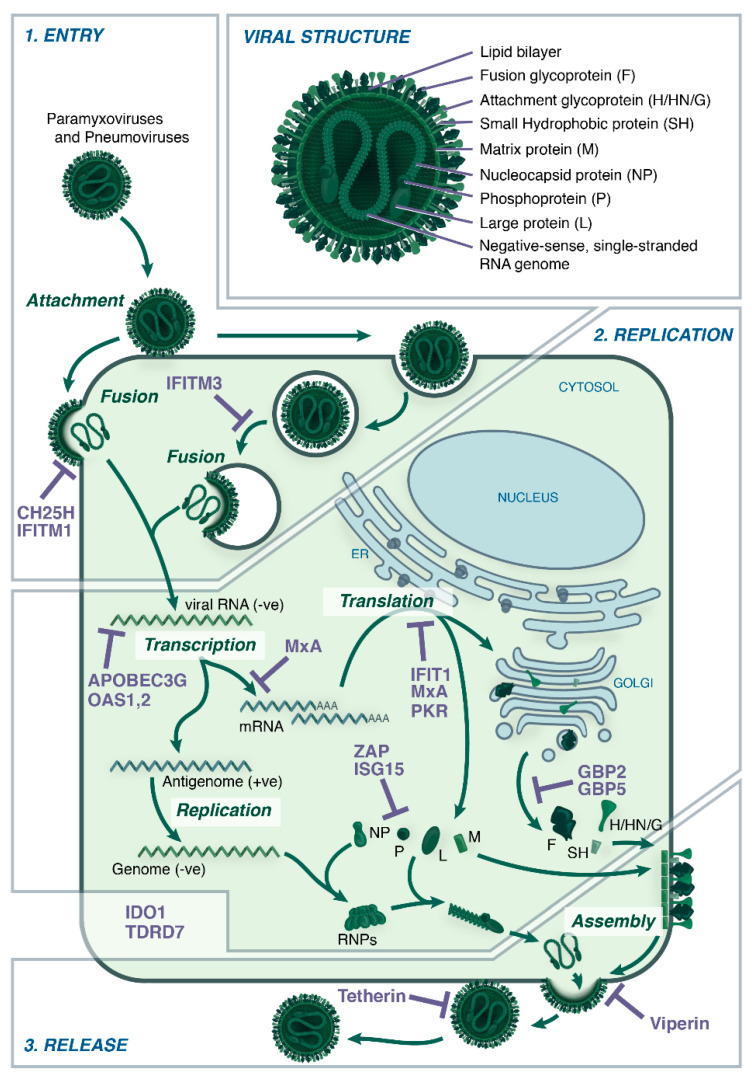
Structure and Replication of Paramyxo- and Pneumoviruses. Paramyxo- and pneumoviruses share a common structure comprising surface fusion (F) and attachment (H/HN/G) proteins (and an additional small hydrophobic (SH) protein for some) located in a host-derived lipid bilayer. Beneath this layer, the matrix (M) protein forms a shell within which is contained the ribonucleocapsid, comprising the phosphoprotein (P) and Large (L) polymerase subunit, as well as a negative-sense, single-stranded RNA genome bound by nucleoprotein (NP) in the form of ribonculeoproteins (RNPs). (1) Upon attachment to the host cell via H/HN/G proteins, paramyxovirus and pneumovirus entry is facilitated by the F protein, releasing the ribonucleocapsid into the cytosol. Pneumoviruses may additionally enter the cell via endocytosis. CH25H, IFITM1 and IFITM3 have been identified to inhibit entry of some viruses. (2) Viral RNA is then transcribed to mRNA and the viral proteins translated, with the glycoproteins (H/HN/G, F and SH) travelling through the ER to the Golgi before reaching the cell surface. Viral RNA is additionally transcribed to produce a positive-sense antigenome, which is replicated to give negative-sense genomic RNA. Restriction factors known to inhibit these stages of virus replication include, ABOBEC3G, OAS1, OAS2, MxA, IFIT1, PKR, ZAP, ISG15, GBP2, and GBP5. The exact step in the virus replication process inhibited by IDO1 and TDRD7 has not been identified. (3) After genomic replication, RNA associates with the NP to form RNPs, as well as the P and L proteins, and traffics to the cell membrane where it interacts with M protein. Once this ribonucleocapsid is assembled with the surface proteins, newly-formed virions are released by budding. The restriction factors tetherin and viperin can interfere with assembly and budding.

Despite comprising a diverse range of viruses, the paramyxo- and pneumoviruses share broad similarities in their virion structure, genome organization, and replication processes (Figure 1) [40]. The outer envelope of the viruses comprises a lipid bilayer derived from the host plasma membranes and at least two viral glycoproteins. While all viruses express a fusion (F) glycoprotein, there are three possible attachment glycoproteins: Haemagglutinin-Neuraminidase (HN, e.g., MuV), Haemagglutinin (HA, e.g., MeV), or Glycoprotein (G, e.g., HRSV) [41]. Some viruses such as HRSV also have a small hydrophobic protein (SH) on the surface, the function of which is still not clear. Underlying the lipid bilayer is the virus matrix protein (M) and enclosed within the virion is a negative (-) stranded genome bound by the nucleocapsid protein (NP) to form helical structures called ribonucleoproteins (RNPs) [41]. The genome codes for six to ten proteins, which includes the glycoproteins (F, HN/H/G, SH), M and NP proteins, a phosphoprotein (P) and a large (L) protein needed for formation of the RNA-dependent RNA polymerase complex (RdRP) [41]. In certain members of the paramyxovirus family, alternate translation initiation or RNA editing of the P gene mRNA can lead to the generation of V, C, or W proteins [40].

Most paramyxo- and pneumoviruses initially infect the airways and transmit via respiratory droplets. Initial binding of virions to respiratory epithelial cells is mediated via the attachment glycoproteins (e.g., HN/H/G) followed by pH-independent fusion with the plasma membrane facilitated by the F glycoprotein, allowing for subsequent release of viral contents into the cytoplasm [41]. Emerging evidence suggests that certain members of these virus families, including NDV, HRSV, and HMPV, can also utilize endocytic pathways to facilitate virus entry [42]. Replication of paramyxo- and pneumoviruses occurs in the cytoplasm, in contrast to influenza A viruses (IAV), where viral transcription and replication occurs in the nucleus [41,43]. The initial phase of replication includes the transcription of (-) stranded genomic RNA to viral mRNA by RdRP, which is then translated into viral proteins. The later phase includes the formation of (+) stranded antigenome, which serves as a template for new (-) stranded genomic RNA for progeny viruses. The virus glycoproteins are processed through the endoplasmic reticulum and Golgi, and then assembled with the M protein at the plasma membrane [41]. The M protein interacts with both the core of RNPs and the cytoplasmic tail of the viral glycoproteins and plays a key role in the final assembly of progeny virions, which are then released by budding [44]. Alongside budding, viruses such as HRSV, NiV, and MeV also employ a second mechanism of viral spread via cell-to-cell fusion between neighboring cells, facilitated by the expression of the F glycoprotein on the surface of infected cells [44]. This leads to the formation of multinucleated syncytia, a well-characterized cytopathic feature of cells infected with HRSV.

## 3. Virus Interactions with the Innate Immune System

During virus replication, paramyxo- and pneumoviruses produce double stranded (ds)RNA and mRNAs without proper 5′ caps that represent pathogen associated molecular patterns (PAMPS) detectable by cell-surface and intracellular pattern recognition receptors (PRRs) [40,45]. At least three major classes of PRRs are known to interact with viral components: Toll-like Receptors (TLRs), Retinoic-acid inducible gene (RIG)-I like receptors (RLR) and NOD-like receptors [45]. Detection of viral PAMPs by these receptors leads to further downstream signaling, eventually leading to induction of type I interferons (e.g., IFN-α, IFN-β). Paramyxo- and pneumoviruses have been shown to mediate type I IFN induction mainly through RIG-I and TLR pathways [1]. Studies with RSV and SeV have also shown that the production of defective viral genomes during viral replication can trigger innate immune signaling, and limit viral replication [46,47].

The RLRs include two RNA helicase proteins, RIG-I and myleoid differentiation association gene (MDA)5, that are expressed in the cytoplasm of most cells and detect dsRNA and 5′pppRNA structures, respectively [40]. Activation of RIG-I and MDA-5 by viral RNA leads to activation of mitochondrial membrane associated adaptor protein (MAVS). Downstream signaling of MAVS activation culminates in the phosphorylation of the cytoplasmic transcription factors interferon regulatory factor (IRF)-3, IRF-7, and nuclear factor κβ (NF-κβ), which translocate to the nucleus and activate transcription of type I IFNs [40].

TLRs are transmembrane proteins that can detect both extra and intracellular viral components. Some TLRs are expressed on the plasma membrane while others are expressed on intracellular endosomal membranes or in the endoplasmic reticulum. TLR3 recognizes dsRNA and is expressed in conventional dendritic cells (DC) and epithelial cells, whereas TLR7 and TLR9 detect ssRNA and are highly expressed in professional IFN-producing plasmacytoid DCs [45]. While TLRs use distinct pathways to those used by RLRs, the signaling converges through the activation of IRF-3, IRF-7, and NF-κβ and the transcription of type I IFNs. The PRRs by which paramyxoviruses are detected by the innate immune system are reviewed in detail elsewhere [40].

Type I IFNs bind to ubiquitously expressed type I IFN receptors (IFNAR) leading to a downstream signaling cascade through the JAK/STAT pathways, which ultimately results in the transcription of hundreds of IFN-stimulated genes (ISGs). Many of these ISGs code for known antiviral proteins that can inhibit the replication of one or more viruses through different mechanisms, and their transcription results in induction of an ‘antiviral state’ in both infected cells and in uninfected neighboring cells [48].

In addition to type I IFNs, type II IFNs (IFN-γ) and type III IFNs (IFN λ) are also important components of the innate antiviral response. Unlike type I IFNs, type II IFNs are produced by only a subset of immune cells (natural killer (NK) cells and activated T cells) and are induced by mitogenic or antigenic stimuli [49]. Type II IFNs have several immunomodulatory and proinflammatory functions, and their antiviral mechanism have overlapping effects with type I IFNs. The antiviral actions of type II IFNs are not particularly well characterized, although their role in viral infections has recently been reviewed in Kang et al. [50]. While type III IFNs (IFN λ) are genetically distinct to type I IFNs, there is a high degree of redundancy in the antiviral responses by type I and type III IFNs (reviewed in [51]).

It should be noted that paramyxo- and pneumoviruses have evolved a variety of mechanisms to antagonize the innate immune response and can therefore reduce the impact of some components of innate immunity. More specifically, the paramyxovirus P/V/C and SH proteins, and the RSV non-structural proteins (NS1 and NS2), have been identified as potent inhibitors of the innate immune system [40,52]. These viral proteins can antagonize the antiviral responses induced in host cells through multiple pathways, including direct antagonism of host cell restriction factors, or interference with antiviral signaling pathways. For example, it has been shown in A549 cells that the HRSV NS1 protein binds to MAVS and prevents interaction with RIG-I, therefore interfering with type I IFN responses by dampening downstream RIG-I signaling [52]. While immune evasion of paramyxo- and pneumoviruses is reviewed in detail elsewhere [1,40], this review will address instances where paramyxo- and/or pneumovirus proteins are known to antagonize particular host cell restriction factors.

## 4. Host Cell Restriction Factors

A range of soluble proteins contribute to the innate host defense against paramyxo- and pneumoviruses, as well as other viral infections, including collectins, defensins, galectins, and complement proteins. However, the largest and most diverse collection of proteins are host cell restriction factors. These are cellular proteins, usually ISGs, which are able to antagonize and limit viral infection, thereby contributing to cell-intrinsic immunity. A number of ISG products, such as myxovirus (Mx) family proteins, 2′-5′ oligo-synthetase (OAS)/RNAseL family proteins, and protein kinase R (PKR), have been particularly well studied and display antiviral activity against a broad spectrum of viruses [48,53]. Other ISGs products have been shown to mediate antiviral activity against one or more viruses, although the specific mechanism/s are often not well defined. Moreover, while hundreds of ISG products are induced in response to viral infection, it is currently unclear if many of these mediate any antiviral activity at all. Cellular host restriction factors may specifically restrict one virus family or display broad spectrum activity against a range of viruses. Moreover, the antiviral mechanisms by which these host proteins act may vary between different viruses. Cellular host restriction factors have been particularly well studied in the context of human immunodeficiency virus (HIV)-1 (reviewed in [54]) and influenza viruses (reviewed in [55]), while less is known regarding their role against other virus families, including paramyxo- and pneumoviruses.

The host cell restriction factors identified for paramyxo- and pneumoviruses to date can be broadly categorized according to which stage of the virus replication they inhibit, even though some may actually impact multiple stages of virus replication (Figure 1). A summary of host cell restriction factors with reported activity against human and animal paramyxo- and pneumoviruses is shown in Table 1. Many of the studies described below utilize a number of approaches to test the antiviral activity of host restriction factors against viruses, with the two most common techniques being: a) overexpression of the protein of interest to test for inhibition of virus infection and/or replication or b) knockdown of the endogenous protein, sometimes following IFN treatment, to test for an enhancement of viral replication.

## 5. Restriction Factors that Affect Viral Entry and Fusion

### 5.1. Interferon Inducible Transmembrane Proteins (IFITM)

The human IFITM family consists of four proteins, IFITM1, IFITM2, IFITM3, and IFITM5, located on chromosome 11, and amongst them, IFITM1, IFITM2, and IFITM3 are well-known known ISG proteins. IFITMs are transmembrane proteins that lack any direct enzymatic activity. While initial studies proposed their structure to comprise a luminal N- and C-terminal domain and a short cytoplasmic domain [56], more evidence suggests an alternative topology whereby the N- and C- terminal domains exhibit a cytosolic orientation [57]. IFITMs display distinct intracellular localization, with IFITM1 expressed in the plasma cytoplasmic membrane, and IFITM2 and IFITM3 localizing to endocytic membranes, including reports describing IFITM2 localization to late endosomes and IFITM3 to early endosomes [16]. IFITM 1, IFITM2, and IFITM3 proteins have a basal expression in variety of cell types in humans but can be strongly upregulated by both type I and II IFNs [56]. IFITM1 has been shown to restrict a number of RNA viruses including Zika virus (ZiV) [58], HIV-1 [59], and IAV [60], amongst others. In vitro over-expression and/or knockdown approaches have shown that IFITM1 inhibits entry of HRSV, HMPV, NDV, PIV-3, and MuV into host cells, with a modest but significant effect also reported against MeV [16,22,61]. As it is widely accepted that paramyxo- and pneumoviruses can enter many cell types via fusion with the plasma membrane, the localization of the IFITM1 protein may be particularly relevant to its reported antiviral activity. Mutations of the conserved intracellular loop (CIL) domain of IFITM1 which altered its localization from the plasma membrane to intracellular structures such as endosomes or lysosomes, reduced antiviral activity against HRSV, MeV, and MuV [16]. Moreover, while IFITM1 overexpression restricted PIV-3 infection, IFITM3 overexpression did not [22]. However, mutation of IFITM3 to achieve expression at the plasma membrane led to inhibition of PIV-3 infection in this study [22]. This provided further evidence that these proteins can modulate paramyxovirus entry at the plasma membrane. The increased disease severity observed in IFITM1-deficient mice following infection with HRSV highlights the ability of IFITM1 to modulate infection in vivo [16].

In contrast to IFITM1, it is well established that IFITM3 can potently inhibit a range of viruses known to enter and infect cells via endocytosis, such as IAV [55], dengue virus (DeV), and West Nile virus (WNV) [60]. IFITM3 has been reported to restrict human pneumoviruses, namely HMPV and HRSV, but not the murine paramyxovirus Sendai virus (SeV) (Table 1) [32,61]. While it is widely accepted that infectious entry of HRSV and HMPV occurs via direct fusion with the plasma membrane, recent evidence indicates these viruses use dual mechanisms of entry and can also utilize endocytic pathways, which explains the inhibition of these viruses by IFITM3 in some cell types [32,62]. While the mechanisms by which IFITM3 can restrict HMPV/HRSV infection has not been fully elucidated, it is potentially similar to its action against IAV, whereby incoming virions are sequestered in endocytic compartments [55]. Bioinformatic analyses and mutational studies show that the membrane modification of these endocytic compartments by the amphipathic helix structure of IFITM3 prevents IAV viral fusion and therefore subsequent release [63]. Studies with HRSV showed that IFITM3 overexpression resulted in delayed phosphorylation of IRF3 and viral RNA interaction with RIG-I and MDA-5, likely due to entrapment of incoming virions within endosomes [61]. The importance of IFITM3 during HRSV infection was reflected in the increased disease severity observed in IFITM3-deficient mice following infection with HRSV [33].

### 5.2. Cholesterol-25-Hydroxylase (CH25H)

CH25H is an endoplasmic reticulum-associated enzyme, conserved across several mammalian species. It has been shown to be upregulated by type I and type II IFNs in murine bone marrow-derived macrophages (BMDM) and DCs, and in response to infection with enveloped viruses such as vesicular stomatitis virus (VSV) [31,64]. This enzyme catalyzes oxidation of cholesterol to produce 25-hydroxycholesterol (25HC), which is then released as a soluble antiviral factor into the medium of infected cells [31]. Treatment of cells with medium containing 25HC has been shown to inhibit replication of a range of DNA and RNA viruses, including the zoonotic paramyxovirus NiV [31]. The product of CH25H, 25HC, appears to exert antiviral activity in a number of different ways that may be distinct for different viruses [65]. For example, it inhibits Zika virus by blocking internalization of the virus [66], but inhibits murine cytomegalovirus (MCMV) and HCV at a post-entry step [67,68]. In the case of NiV, transfection experiments in Vero cells showed that 25HC inhibited viral fusion to the cell membrane, therefore blocking virus entry into cells. Further analysis indicated that 25HC directly modified the properties of the cell membrane, which contributed to its antiviral activity against NiV [31]. However, further in vivo analysis was not done to confirm the antiviral role of CH25H against NiV.

Over-expression and knockdown experiments have also shown that the CH25H enzyme inhibits bovine (B)PIV-3 replication [65]. While 25HC was required for its antiviral activity, modification of the enzymatic domain of CH25H did not fully abrogate its antiviral capacity. This suggests that CH25H may also exert antiviral activity independent of its enzymatic product 25HC. Further to this, by testing different stages of viral replication (i.e., attachment, internalization, replication, and release), it was shown that CH25H inhibited synthesis of viral (+) genomic and (-) anti-genomic RNA, therefore acting at the replication stage of infection [65]. Further studies are required to fully elucidate the mechanisms by which CH25H mediates antiviral activity against different viruses.

## 6. Restriction Factors that Affect Virus Transcription, Translation, or Protein Synthesis

### 6.1. IFN-Induced Proteins with Tetratricopeptide Repeats (IFIT)

Human IFIT family proteins are comprised of four members: IFIT1, IFIT2, IFIT3, and IFIT5, while only IFIT1, 2, and 3 are present in mice. The IFIT proteins lack enzymatic activity and are localized within the cytoplasm. They are strongly induced in response to virus infection or exogenous treatment with IFNα [56]. The IFIT family has broad-spectrum antiviral activity against many viruses belonging to the Flavi-, bunya-, rhabo- and orthomyxovirus families [56]. Their antiviral activity can be mediated by inhibiting the initiation of translation through binding the subunits of the eukaryotic initiation factor 3 (eIF3) translation initiation complex. IFITs are also capable of binding and sequestering ‘non-self’ viral mRNA, often characterized by a lack of 2′-O methylation or uncapped 5′-ppp. Studies with PIV-5, with a mutated non-functional V protein that antagonizes IFN signaling, demonstrated that IFIT1 was a potent inhibitor of this virus [23]. While viral mRNA levels were not affected by over-expression of IFIT1, protein synthesis was reduced, suggesting inhibition of mRNA translation [18,23]. Other members of the rubulavirus subfamily of paramyxoviruses, namely PIV-2 and MuV, were also sensitive to the antiviral effects of IFIT1, whereas PIV-3, SeV, and CDV were not. This suggests that IFIT1 can specifically recognize the RNA of rubulavirus subfamily viruses within the paramyxovirus family [18]. Further mechanistic insights regarding IFIT1-mediated antiviral activity were not provided in these studies, although it was noted that the PIV-5 NP protein, which is normally present throughout the cytoplasm, relocated with cytoplasmic inclusion bodies following IFIT1 overexpression [23].

Chicken IFIT5 (chIFIT5), the only IFIT protein encoded by chickens, was shown to be induced in chicken fibroblasts in response to NDV infection [28]. The antiviral role of chIFIT5 was confirmed by inhibition of viral growth in cells with inducible over-expression of chIFIT5 or by enhancement of viral growth following CRISPR/Cas9 knockout of chIFIT5 in chicken fibroblasts. Similar overexpression and knockdown approaches in chicken embryos confirmed the relevance of these results in vivo. Further studies showed that chIFIT5 was able to uniquely identify NDV mRNA expressing a 5′-ppp motif; however, further details on possible mechanisms of antiviral action were not detailed [28].

### 6.2. Myxovirus Resistance (Mx) Proteins and Other GTPases

Mx proteins of the GTPase superfamily are potently induced in response to type I and type III IFN signaling [69]. Humans and mice each express two Mx proteins, MxA/MxB and Mx1/Mx2, respectively. Human MxA, which localizes to the cytoplasm, has antiviral activity against a broad range of RNA and DNA viruses, including IAV [55,70] and Hepatitis B virus (HBV) [71], while human MxB, which localizes to the nucleus, has antiviral activity against HIV-1 [72]. MxA can inhibit viral infection at multiple steps of the replication cycle. A proposed model of MxA antiviral action suggests the formation of ring-like oligomeric structures that trap viral components and prevent replication. For example, human MxA can trap Thogoto virus or IAV nucleocapsids and prevent primary transcription of viral RNA but can also sequester the N protein of bunyaviruses such as La Crosse Virus (LACV) and prevent the viral polymerase from mediating genomic replication [69].

With regard to paramyxoviruses, the antiviral activity of human MxA against MeV was demonstrated in 1994, where transfection of the human monocytic U937 cell line to overexpress MxA resulted in reduced titres of infectious virus released from MeV-infected cells [10]. This study showed that inhibition occurred at a post-transcriptional stage, and that viral F and H glycoprotein synthesis were targeted by MxA [10]. In contrast, a later study showed that MxA-dependent antiviral activity against MeV in human brain cells was mediated by restriction of genomic transcription [73]. Together, these studies suggest that MxA mediates antiviral activity by distinct mechanisms in different host cell subsets. Genome association studies in humans further highlight the importance of MxA in persistent MeV infections, as SNPs in the MxA promoter region are associated with subacute sclerosing panencephalitis [74].

MxA has also been shown to affect PIV-3 and PIV-5 replication. For PIV-3, MxA appears to exert its antiviral effect during the early stages of replication, just after primary transcription [20], while for PIV-5 the latter stages of replication are inhibited at a post-translational step [75]. While in vitro experiments show MxA does not inhibit HRSV replication [76], it is likely that MxA may still play a role in vivo, as polymorphisms in the MxA gene have been linked to severe HRSV infections in children [77].

Guanylate-binding proteins (GBPs) of the GTPase superfamily are induced in response to type I and type III IFNs and can display antiviral activity against some viruses. Humans express seven GBP proteins (GBP1-7), and GBP1 has been shown to restrict VSV, encephalomycarditis virus (EMCV) and HCV [78,79]. Compared to Mx proteins, less is known about the antiviral activities of GBPs. Recently, over-expression experiments demonstrated that GBP2 and GBP5 were capable of inhibiting HIV-1 infectivity [13]. Further analysis demonstrated that GBP2 and GBP5 bound to the cytoplasmic domain of furin, a protease responsible for maturation of the HIV-1 envelope (Env) glycoprotein and reduced its proteolytic activity. Subsequent experiments confirmed antiviral activity against a range of additional viruses which depend on furin-mediated processing of glycoproteins, including MeV [13]. Interestingly, another recent study showed that human GBP5, largely induced by type III IFNs, was able to inhibit HRSV replication through an alternate mechanism. This study demonstrated that GBP5 downregulated secretion of RSV SH protein, independent of its catalytic activity [80]. The antiviral inhibition of GBP5 could be overcome by exogenous supplementation of viral SH protein. The study further showed that the RSV-G protein antagonizes this antiviral activity by downregulating GBP5 expression through the proteasomal pathway, via upregulation of the E3 ligase, DZIP3 [80].

In addition to studies with human GBPs, mouse GBP4 was shown to inhibit SeV infection by a completely different mechanism. Overexpression of mGBP4 inhibited SeV-induced IFN-sensitive response element [ISRE] signaling. Mouse GBP4 was shown to interact directly with IRF7, a transcription regulator for IFN signaling, which disrupted the association between TRAF6 and IRF7 required for IRF7 phosphorylation [29]. Currently, no unifying mechanisms of GBP antiviral action are apparent and a systematic investigation is required to assess the antiviral activity and the mechanisms by which GBPs can inhibit different viruses, including paramyxo- and pneumoviruses.

### 6.3. 2′-5′ Oligosynthetase Proteins (OAS)

The OAS proteins are IFN-inducible ISG proteins that belong the nucleotydilytransferase enzyme family. There are four human OAS proteins (OAS-1,2,3, and L) and six in mice (OAS-1,2,3,4,L1, and L2), but the human OASL and the mouse OASL1 proteins lack any catalytic activity [48]. The canonical function of OAS proteins is to synthesise 2′-5′ phosphodiester bonds between ATPs, leading to the formation of 2′-5′A linked oligomers, which in turn activate the RNaseL enzyme. The RNaseL enzyme can then degrade viral RNA, and therefore the OAS/RNaseL pathway can mediate its antiviral action by preventing transcription and replication of viral RNA. Degraded viral RNA is also detected by the RIG-I and MDA-5 PRRs, which sets up a positive feedback loop for increased IFN induction [48,81]. The antiviral function of OAS proteins have been demonstrated in RNaseL-deficient mice, which display increased susceptibility to viruses from multiple families including, *Picornaviridae*, *Orthomyxoviridae*, *Flaviviridae*, *Reoviridae*, and *Togaviridae* [48].

In the human A549 lung and Hep2 laryngeal epithelial cell line lines, IFN-γ treatment resulted in both reduced HRSV viral titres and accumulation of OAS1 and OAS2 mRNA levels [35], suggesting a role for the OAS/RNaseL pathway in restriction of HRSV infection [35]. IFN-γ-induced RSV inhibition was ablated using antisense oligonucleotides against OAS1 and OAS2, or RNAseL inhibitors in HEP2 cells, confirming the role of this degradation pathway in viral restriction [35]. In African Green monkeys infected with HRSV, treatment with antisense compounds to OAS1 and 2 reduced viral titres approximately 10,000-fold compared to saline-treated animals at day 6 post-infection [82].

The OAS proteins can also inhibit viruses independent of catalytic activity, as stable overexpression of OASL in HEK293 cells has been shown to inhibit SeV and HRSV replication [83,84]. These overexpression results were confirmed by knockdown of OASL in these cells, which resulted in enhanced viral replication. Further analysis revealed that the C-terminal ubiquitin-like domain of the OASL protein interacts with the RIG-I caspase-recruitment domain (CARD) and therefore increases RIG-I-mediated antiviral signaling, which in turn results in inhibition of viral replication [83,84]. However, the HRSV NS1 protein is able to suppress expression of OASL, thereby antagonizing OASL-mediated virus restriction [83]. Goose OASL, which has a catalytic domain, has also been implicated in the restriction of avian viruses, including NDV and IAV (H9N2). Structural analysis of goose OASL suggests it may inhibit infection through both RNaseL-dependent RNA degradation pathway and also RNase L-independent (RIG-I signaling) pathways [25].

### 6.4. Apolipoprotein B (apoB) mRNA-Editing Enzyme Catalytic Polypeptide (APOBEC) Proteins

The APOBEC family proteins are IFN-inducible cytidine deaminases, with 11 members of the human APOBEC family reported to date. APOBEC3G (A3G) is the best characterized of these proteins, after it was discovered to exhibit antiviral activity against HIV-1 in 2002 [85]. The antiviral activity of A3G can be mediated through its deaminase activity which introduces hypermutations into the viral genome. However, A3G can also inhibit viral replication in a deaminase-independent manner, whereby it binds directly to viral mRNA and prevents transcription [86]. Experiments in Vero cells have shown that A3G is able to restrict members of the paramyxo- and pneumovirus families, including HRSV, MeV, and MuV [11]. Using MeV as a model virus, A3G overexpression resulted in modest reductions in viral mRNA levels (50–60% compared to controls), partially explaining its antiviral mechanism. Serial passaging of MeV in cells overexpressing A3G also resulted in accumulation of mutations in the viral genome at a much higher frequency than in control cell lines. This demonstrated an alternative mechanism of antiviral action of A3G, though it should be noted that the nature of the mutations introduced suggested they were not dependent on its deaminase activity [11]. Chicken APOBEC4G, which shares structural similarities to human A3G, has been shown to be upregulated in vivo in response to NDV infection. Further in vitro analyses demonstrated that this protein reduced transcription of NDV viral RNA, and therefore titres of virus released from NDV-infected cells [27].

### 6.5. Indoleamine 2,3-Dioxygenase (IDO)

IDO1 is an immunomodulatory heme containing enzyme, that catalyzes the first rate-limiting step of the kyrunenine pathway leading to tryptophan degradation. IDO1 is typically induced in response to IFN-γ, and has antiviral activity against human cytomegalovirus (HCMV) [87] and herpes simplex virus (HSV)-1 [88]. The antiviral activity of IDO1 has also been demonstrated against PIV-3, MeV, and HRSV [14,22,34,89]. Addition of exogenous tryptophan abrogates the antiviral activity of IDO1 against MeV and PIV-3, suggesting that tryptophan depletion may be its primary mechanism of antiviral activity [14,22]. While these studies were performed using human epithelial cell lines treated with IFN-γ, it was found that HRSV infection of human mesenchymal stromal cells (MSC) led to IDO1 induction through upregulation of IFN-β rather than IFN-γ [89]. Despite results from in vitro studies, virus titres in the lungs of HRSV-infected IDO1 deficient mice were similar to those of control animals five days post-infection [89]. This result was partially explained by a follow-up experiment that showed that, unlike human mesenchymal stromal cells, IDO1 was not induced in mouse mesenchymal stromal cells by IFN-β. Therefore, biological differences between human and mouse mesenchymal cells may have contributed to the lack of in vivo effect seen with IDO1 [89].

### 6.6. Protein Kinase R (PKR)

Double-stranded RNA-dependent PKR is a classic ISG protein that was initially reported to restrict vaccinia virus (VV) by inhibiting translation of viral and cellular proteins from mRNA [90]. The mechanism by which PKR inhibits translation is via the phosphorylation and inactivation of eukaryotic translation initiation factor 2 α (eIF2α) [90]. In addition, PKR is involved in upregulating type I IFN induction by activating NF-κβ and enhancing IFN-β transcript levels [90]. Inactivation of eIF2α leads to the formation of stress granules, which store inactive mRNA [15]. PKR-dependent stress granule formation has been observed in cells infected with MeV and HRSV; however, HRSV viral titres were not affected by knockdown of PKR [15,37]. MeV lacking the viral C protein were more efficient in forming stress granules, suggesting this protein plays a key role in antagonizing PKR antiviral activity [15]. Similarly, PKR activation was observed in SeV virus lacking the C protein, which strongly antagonizes IFN responses [91]. A combination of inhibition and overexpression studies also showed that PKR moderately restricted PIV-3, but not PIV-5 infection in vitro [22,75]. Despite well-characterized antiviral activity against other viruses, there is relatively little detail regarding PKR-mediated inhibition of paramyxoviruses.

### 6.7. Interferon Stimulated Gene 15 (ISG15)

ISG15 is a 15kDa protein, similar to ubiquitin, which through conjugation to multiple protein targets (ISGylation), regulates their function within host cells. ISG15 is strongly induced in response to type I and type III IFNs and has been shown to mediate pro-viral [92] and antiviral effects [93] through modulation of specific cellular proteins. Protein purification assays from IFNβ-treated Hela cells have identified up to 158 putative target proteins for ISG15 [94]. A number of these proteins are constitutively expressed and involved in diverse cellular pathways such as RNA splicing, cytoskeleton organization and regulation, stress responses, and translation. However several target proteins were also ISG proteins themselves, or part of the type I IFN signaling pathway, and examples include PKR, MxA, HuP56, and RIG-I [94]. The ISGylation of proteins is mediated via a sequential reaction involving E1 activating (UbEL1), E2 conjugating (UbcH8), and E3 ligating (HERC5) enzymes [95,96].

Microarray analysis demonstrated strong upregulation of ISG15 in HRSV-infected A549 cells [97]. Subsequent in-depth analysis utilising over-expression and knockdown assays confirmed the ability of ISG15 to inhibit HRSV growth and that it functioned at a post-entry stage of HRSV infection [36]. This study also confirmed that protein ISGylation was required for ISG15 mediated inhibition of HRSV, though the specific target proteins and their action were not defined [36]. In contrast to HRSV, ISG15-deficient cells were shown to be resistant to infection by paramyxoviruses such as SeV, NiV and PIV-5, suggesting a pro-viral role for ISG15 to facilitate efficient cellular infection [98,99]. The mechanisms underlying the contrasting role of ISG15 during paramyxo- and pneumoxovirus infections are yet to be elucidated.

### 6.8. Other Host Restriction Factors

Zinc antiviral protein (ZAP) is another broad-spectrum antiviral ISG protein that has been shown to restrict a wide range of viruses including IAV, HBV, alphaviruses, and reoviruses. There are two isoforms of ZAP; ZAP-S for the shorter isoform and ZAP-L for the longer isoform. The antiviral action of ZAP is multi-faceted, whereby it can bind directly to viral RNA and promote its degradation, or block translation by interfering with translation initiation factors eIF4G and eIF4a [53]. In a recent meeting report, ZAP-L (but not ZAP-S) was reported to bind to the matrix protein of multiple paramyxoviruses and restrict their replication, with the exception of SeV [100]. However, very little detail is available from this yet-to-be published study, including the specific paramyxoviruses tested in their panel.

Tumor domain containing 7 (TDRD7) was recently discovered through a shRNA lentiviral-based knockdown screening assay with SeV [21]. TDRD7 was also shown to be induced in response to IFN-β and other external stimuli such as poly I:C. Subsequent overexpression and knockdown studies confirmed its ability to restrict SeV, HRSV, and PIV-3. Further analysis showed that TDRD7 inhibited viral-induced autophagy, which is critical for viral replication, by interfering with activation of autophagy-induced AMP-dependent kinase [21]. Another restriction factor, caspase recruitment domain family member 11 (CARD11), was discovered by microarray analysis on NDV-infected chicken brains [26]. Overexpression of CARD11 in chicken neuronal cells reduced NDV infection, while knockdown increased viral replication. CARD11 was shown to interact with the NDV P protein by co-immunoprecipitation and to reduce the polymerase activity of the viral RNP complex [26]. Widespread ISG screening assays by Schoggins et al. have also identified known antiviral genes such as TRIM25 and HPSE to be upregulated in response to RSV, HMPV, and MeV infections; however, their protein products were not characterized further in this study [101].

Finally, it should be noted that host non-coding (nc)RNAs have recently been implicated in host defense and antiviral action in response to viral infections. MicroRNA (ss 18-24 length host RNA) expression profiles in airway epithelial cells are dramatically changed in response to HRSV and HMPV infections [102]. Some examples of miRNAs implicated in host defense include (i) mIR-140-5p, which assists with TLR signaling and inhibits growth of viruses such as HRSV, (ii) Let-7f, which inhibits HMPV but not HRSV and (iii) gga-miR-455-5p, which suppresses NDV replication [102,103]. NcRNA and their role in cellular defense against HRSV and HMPV has been reviewed elsewhere [102].

## 7. Restriction Factors that Affect Virus Release

### 7.1. Viperin

Viperin is an IFN-inducible protein that restricts a number of different viruses at a late stage of the virus replication cycle. It has been shown to localize to lipid droplets in the plasma membrane and to inhibit the enzyme farnesyl diphosphate synthetase [104]. This, in turn, disrupts the lipid raft domains and interferes with budding of viruses such as HIV and IAV [53,104]. However, viperin has also been shown to co-localize to lipid droplets with viral proteins, as is seen with DeV, suggesting it has varying mechanisms of antiviral action against different viruses [105].

In response to HRSV infection, viperin is upregulated in chinchilla nasopharynx [38] and in mouse lungs [106]. It is also upregulated in the blood of human volunteers infected with HRSV [107]. Over-expression of human viperin in chinchilla airways following recombinant adeno-associated virus (rAAV) vector transduction led to reduced titres of HRSV in the nasal turbinates. This experiment confirmed the inhibitory role of viperin during HRSV infection [38]. Of note, however, knockdown studies were not performed to clarify the role of endogenous viperin in HRSV inhibition. While mechanistic studies were not performed, the authors speculated that viperin-mediated restriction of HRSV might relate to its ability to disrupt lipid raft domains [38]. Viperin overexpression was also shown to restrict the growth of MeV and PIV-3 in vitro, although for MeV, only titres of cell-free, but not cell-associated virus, were reduced [12,22]. Mutation of residues in the N- and C-terminal, or in the central Fe-S cluster domain of viperin, confirmed that all three domains were required for restriction of MeV. Beyond this, the mechanisms by which viperin inhibits MeV release have not been defined [12].

### 7.2. Tetherin

Tetherin is a type II transmembrane protein that was first identified as a key antiviral factor against HIV-1 [108]. It exerts its antiviral action by physically tethering virus particles to the cell surface, thereby preventing viral release [109]. It is able to do so by interacting with the cell membrane using its N-terminal transmembrane domain and with the viral lipid bilayer using its C-terminal glycosylphosphatidylinositol (GPI) anchor [109]. While tetherin has been shown to restrict a wide array of enveloped viruses, including HIV-2, simian immunodeficiency virus (SIV), Ebola virus, Herpes virus, and Lassa virus, there are also a number of well-known viral antagonists to tetherin [110]. The HIV-1 Vpu protein, the envelope proteins of SIV and Ebola, the Nef protein of SIV, and the K5 protein of Kaposi’s sarcoma-associated herpesvirus are known to overcome tetherin restriction by different mechanisms [110].

Utilising virus-like particles (VLPs) containing the M protein of NiV and SeV, it was shown that tetherin was able to restrict the budding of these viruses. Co-transfection and microscopy confirmed that tetherin and NiV-M accumulated at the plasma membrane. Interestingly, MuV-M VLPs were not affected by tetherin, suggesting differences in the M protein of paramyxoviruses alone could lead to varying levels of tetherin restriction [30]. Co-transfection of tetherin with HIV-1 Vpu did not abrogate the inhibition of NiV-M and SeV-M by tetherin. In a separate study, human and murine tetherin expression was also shown to inhibit VLPs containing NiV-M protein, as well as members of the arenavirus family, but not filoviruses [111]. However, the antiviral role of tetherin against these paramxyoviruses has not yet been validated in vivo.

## 8. Perspective and Conclusions

The field of host cell restriction factors is most well studied in regard to HIV-1 [54] and IAV [55] and, as such, details regarding restriction factors active against other viruses, including paramyxo- and pneumoviruses, are lacking. Within these virus families, HRSV, MeV and SeV are most well studied, whereas details on restriction on other family members such as HMPV, NiV or MuV are more spurious. Given the combined disease burden of these virus families in regard to global health, there is a further need to study host restriction factors against these lesser known family members.

Many restriction factors identified against paramyxo- or pneumoviruses are well-characterized ISG proteins with broad antiviral activity, such as MxA or OAS. Identification of ISGs has traditionally relied on gene microarray analysis or expression studies, as performed for HRSV [112] and MeV [113]. However, more sophisticated screening assays employing shRNA-based lentiviral knockdowns of specific genes as was recently reported using SeV [21] or using CRISPR-based technology for HIV-1 [114]. Lentiviral-based ISG expression assays combined with flow-cytometry based screening have also been developed and used to identify inhibitory ISG panels for multiple DNA and RNA viruses [101]. These high-throughput techniques could play an important role in identifying novel restriction factors for lesser studied viruses in the future. Comparison analysis of the differential responses between non-permissive immune cells, such as airway macrophages, compared to permissive epithelial cells, has been reported for IAV [105] and could also be a useful approach to exploit for the identification of restriction factors against paramyxo- and pneumoviruses. In addition to in vitro analyses, advances in human genomics is an exciting frontier for exploring the role of restriction factors in disease development. Much information can be gleaned from the increasingly common genome-wide association studies, which can link polymorphisms in specific genes with severity of infections.

The study of host restriction factors within the context of a virus infection often relies on in vitro experiments utilizing transient transfection-based assays to confirm inhibition of viral growth. However, the physiological relevance of these approaches can sometimes be quite low. Often, in vitro studies use non-polarized epithelial cell-lines while most in vivo studies rely on animal models semi-permissive for infection, such as observed with HRSV in mice. Although these approaches have revealed important insights regarding viral infection, replication, and associated inflammatory responses, they do not broadly recapitulate the early interactions and potential consequences of infection of the human columnar airway epithelium in vivo. Hence, it is important to further develop and utilize primary airway epithelial cell culture techniques, including at the air-liquid interface, to better understand these virus-host interactions. In vitro studies may also fail to capture the complex roles that a particular antiviral protein may play in restricting viral infection. For example, early studies dismissed MxA as a potential restriction factor for HRSV; however, it may still hold physiological relevance as polymorphisms in this gene are associated HRSV disease severity. It should be noted that for some of the host restriction factors mentioned in this paper, such as CH25H, ISG15 and tetherin, in vivo studies confirming their antiviral roles have not yet been performed. Another complicating factor in studying host restriction, is the strong antagonism displayed by certain virus proteins to IFN signaling, as has been observed with the NS1 and NS2 proteins of HRSV, or the V protein of PIV-5. The exact mechanistic detail of several of the host cell restriction factors discussed in this review, including viperin, ISG15 or CH25H is still lacking, and is confounded by their potential to act at multiple stages of the virus replication cycle. A further complicating factor is that the mechanism of action may vary between host cell subsets. This was observed with reported differences in the antiviral activity of MxA against in MeV in human monocytic cells versus human brain cells. It is also curious to note that despite the general structural similarity between members of the paramyxoviruses, certain restriction factors such as IFIT1 only appear to inhibit a subgroup of these viruses, the rubulaviruses, but not others [18].

Alongside identification and characterization of host factors for different virus families, exploring therapeutics that can modify their expression is an important avenue for further research. An ideal therapeutic would target a broad range of viruses; however, this could be difficult due the great diversity observed amongst respiratory viruses. For example, orthomyxoviruses such as IAV have a segmented negative-stranded RNA genome and coronaviruses express a positive-stranded non-segmented genome, whereas the genome of paramyxo- and pneumoviruses is comprised of non-segmented negative-stranded RNA. The viral replication cycle can also vary significantly, with IAV entering the cells via endocytosis, replicating in the nucleus and budding from the cell surface, while paramyxo- and pneunoviruses can also enter via fusion with the plasma membrane, replicate within in the cytoplasm and some spread via cell-to-cell fusion. This could make it difficult to identify any one ISG protein that can overcome the differential properties of these viruses and mediate potent and broad activity against different respiratory viruses.

Nevertheless, therapeutics that can target the innate immune system is an expanding area of research. For example, a number of RNA-based or small molecule compounds that act as RIG-I agonists are being developed as pan-viral therapeutics [115]. A recent study showed that a small molecule compound, hydroxyquinolines, inhibited the growth of viruses from *Flaviviridae, Filoviridae, Orthomyxoviridae, Paramyxoviridae*, and *Arenaviridae* families, in vitro [116]. Similarly TLR agonists can also have antiviral potential with candidate molecules having shown activity against HIV-1 (GS-9620) [117], HBV (GS-9620) [118] and IAV [119]. While RIG-I and TLR agonists target the IFN signaling pathway, candidates that target ISGs can also be promising, though there is less research in this area. Currently, most research focuses on identifying ISGs and elucidating their mechanism of action. However, as demonstrated with anti-OAS RSV compounds, the findings from in vitro studies have the potential to be scaled to translational products. Advances in our knowledge and understanding of restriction factors remain important as they can pave the path towards the development of broad-spectrum antivirals, addressing the unmet need for newer therapeutics against respiratory viruses.

## Figures and Tables

**Table 1:** Host cell restriction factors with reported activity against paramyxoviruses and pneumoviruses.

Virus	Genus	Species	Disease	Vaccines and Antivirals	Restriction Factors
***Paramyxoviruses***					
**MeV**	Morbillivirus	Human	Systemic	MMR (measles-mumps-rubella) vaccine, No licensed antiviral [9]	MxA ([10], Apobec3g [11], Viperin [12], GBP2 and 5 [13], IDO1 [14], PKR [15], IFITM1 [16]
**MuV**	Orthorubulavirus	Human	Systemic	MMR (measles-mumps-rubella) vaccine, No licensed antiviral [17]	IFITM1 [16], Apobec3g [11], IFIT1 [18]
**PIV-2**	Orthorubulavirus	Human	Respiratory	No licensed vaccine or antiviral [19]	IFIT1 [18]
**PIV-3**	Respirovirus	Human	Respiratory	No licensed vaccine or antiviral [19]	MxA [20], TDRD7 [21], PKR [22], IDO1 [22], IFIT1 [22]
**PIV-5**	Orthorubulavirus	Human	Respiratory	No licensed vaccine or antiviral [19]	IFIT1 [23]
**NDV**	Orthoavalovirus	Avian	Systemic	Inactivated or Live vaccine available [24], No licensed antiviral	OASL (avian) [25], CARD11 ([26], Apobec4g (avian) [27], IFIT5 (Avian) [28], IFITM1 [16]
**SeV**	Respirovirus	Mouse	Respiratory	No licensed vaccine or antiviral	TDRD7 [21], GBP4 (mouse) [29], Tetherin [30]
**NiV**	Henipavirus	Fruit bats/Human (zoonotic)	Systemic	No licensed vaccine or antiviral	Tetherin [30], CH25H [31]
***Pneumoviruses***					
**HMPV**	Metapneumovirus	Human	Respiratory	No licensed vaccine or antiviral [19]	IFITM1 [16], IFITM3 [32]
**HRSV**	Orthopneumovirus	Human	Respiratory	No licensed vaccine, Ribavarin approved for severe infections, Palivizumab (monoclonal antibody) approved as prophylaxis for high risk infants and children [19]	IFITM1 [16], IFITM3 [33], IDO1 [34], OAS [35], TDRD7 [21], ISG15 [36], Apobec3g [11], PKR [37], Viperin [38]
